# Indium-111 radiolabelling of a brain-penetrant Aβ antibody for SPECT imaging

**DOI:** 10.48101/ujms.v129.10585

**Published:** 2024-05-20

**Authors:** Tobias Gustavsson, Matthias M. Herth, Dag Sehlin, Stina Syvänen

**Affiliations:** a Department of Public Health and Caring Sciences, Uppsala University, Uppsala, Sweden; b Department of Drug Design and Pharmacology, University of Copenhagen, Copenhagen, Denmark

**Keywords:** SPECT imaging, Amyloid-beta, antibody, Indium-111

## Abstract

**Background:**

The development of bispecific antibodies that can traverse the blood–brain barrier has paved the way for brain-directed immunotherapy and when radiolabelled, immunoPET imaging. The objective of this study was to investigate how indium-111 (^111^In) radiolabelling with compatible chelators affects the brain delivery and peripheral biodistribution of the bispecific antibody RmAb158-scFv8D3, which binds to amyloid-beta (Aβ) and the transferrin receptor (TfR), in Aβ pathology-expressing tg-ArcSwe mice and aged-matched wild-type control mice.

**Methods:**

Bispecific RmAb158-scFv8D3 (biAb) was radiolabelled with ^111^In using CHX-A”-DTPA, DOTA, or DOTA-tetrazine (DOTA-Tz). Affinity toward TfR and Aβ, as well as stability, was investigated *in vitro*. Mice were then intravenously administered with the three different radiolabelled biAb variants, and blood samples were collected for monitoring pharmacokinetics. Brain concentration was quantified after 2 and 72 h, and organ-specific retention was measured at 72 h by gamma counting. A subset of mice also underwent whole-body Single-photon emission computed tomography (SPECT) scanning at 72 h after injection. Following post-mortem isolation, the brains of tg-ArcSwe and WT mice were sectioned, and the spatial distribution of biAb was further investigated with autoradiography.

**Results:**

All three [^111^In]biAb variants displayed similar blood pharmacokinetics and brain uptake at 2 h after administration. Radiolabelling did not compromise affinity, and all variants showed good stability, especially the DOTA-Tz variant. Whole-body SPECT scanning indicated high liver, spleen, and bone accumulation of all [^111^In]biAb variants. Subsequent *ex vivo* measurement of organ retention confirmed SPECT data, with retention in the spleen, liver, and bone – with very high bone marrow retention. *Ex vivo* gamma measurement of brain tissue, isolated at 72 h post-injection, and *ex vivo* autoradiography showed that WT mice, despite the absence of Aβ, exhibited comparable brain concentrations of [^111^In]biAb as those found in the tg-ArcSwe brain.

**Conclusions:**

The successful ^111^In-labelling of biAb with retained binding to TfR and Aβ, and retained ability to enter the brain, demonstrated that ^111^In can be used to generate radioligands for brain imaging. A high degree of [^111^In]biAb in bone marrow and intracellular accumulation in brain tissue indicated some off-target interactions or potential interaction with intrabrain TfR resulting in a relatively high non-specific background signal.

## Introduction

Alzheimer’s disease (AD) is the primary form of dementia, contributing to 70–80% of all dementia cases and is globally projected to increase from 45 million to 150 million cases by 2050 (1). Until recently, no disease-halting treatment has been available, besides symptomatic alleviation with cholinesterase inhibitors and N-methyl-D-aspartate (NMDA) receptor antagonists. In 2023, two decades since the introduction of the last new symptomatic treatment, the antibody *lecanemab* was approved by the American Drug and Food Agency (FDA) as a disease-modifying immunotherapy after showing effects on both amyloid-beta (Aβ) brain levels and cognitive performance among AD patients (2). In addition, two other anti-Aβ antibodies, *Aducanumab* (3, 4) and *Donanemab* (5), have also shown similar effects in late-phase clinical trials. Historically, in line with other complex brain diseases, a major hurdle in the development of new treatments for AD has been the lack of available biomarkers for studies of pathology progression and potential drug effects on pathology. In the field of AD, increased availability of medical imaging such as positron emission tomography (PET) has improved the possibilities to assess Aβ levels in the living brain. Especially the use of PET radioligands that bind to amyloid present in the core of the Aβ aggregates has been important for the emerging therapeutic Aβ antibodies (2–6).

According to the amyloid cascade hypothesis, the accumulation and deposition of Aβ are the initial triggers that facilitate pathological changes in the AD brain, including neurofibrillary tangles of hyperphosphorylated tau and neuroinflammation (7). Although insoluble Aβ fibrils that form amyloid are the main constituents in plaques, smaller soluble and diffuse Aβ aggregates are linked to AD progression and severity (8, 9). For example, *lecanemab* is primarily directed toward protofibrillar Aβ (6, 10). Thus, despite the successful use of brain imaging in the recent development of antibody-based therapies, there is a mismatch between the imaging target and treatment target (11).

One strategy to enable imaging of prefibrillar Aβ aggregates is to use the antibodies also as radioligands (12–16). However, brain entry of macromolecules, including antibodies, is severely limited due to the blood–brain barrier (BBB), a capillary structure composed of tightly connected endothelial cells, pericytes, and astrocyte endfeet projections that form a protective barrier in the brain. Nonetheless, the brain requires specific circulation-derived proteins. One example is endogenous transferrin, which carries iron and is transported across the BBB by the transferrin receptor (TfR). Due to its ‘shuttle characteristics’, TfR has also been targeted to enhance brain uptake of engineered proteins, including antibodies (14, 17–21).

We have previously created a bispecific variant of mAb158, which is the murine parent version of *lecanemab*, by recombinant fusion of a single-chain variable fragment (scFv) of a mouse TfR (mTfR) targeting antibody, 8D3 (22), to the C-terminal light chains of mAb158, forming RmAb158-scFv8D3 (17). This bispecific antibody, from now on referred to as biAb, displays rapid brain uptake and uniform spatial distribution in brain areas that harbor Aβ pathology (21, 23). Furthermore, when radiolabelled with the PET radionuclide iodine-124, [^124^I]biAb can be used as a PET radioligand in Aβ mouse models to quantify brain Aβ levels at different pathology stages and to detect changes in brain Aβ levels following BACE inhibition treatment (12, 13). The SPECT is a somewhat less expensive technique than PET and is more frequently available. Iodine-125 is a radionuclide that is compatible with SPECT, and studies using [^125^I]biAb have demonstrated that also this SPECT radioligand can detect brain Aβ (23).

As exemplified above, the choice of radionuclide and its decay type determine the imaging modality, that is, β^+^- or γ-emission for utilization in diagnostic PET and SPECT imaging, respectively. The choice of radionuclide dictates labelling chemistry for successful protein labelling. Proteins can be directly labelled with chloramine-T that facilitates radioiodination of tyrosine residues in the protein (24). This strategy is relatively straightforward but not widely used clinically, as free radioiodine, generated *in vivo* during the degradation of the radioligand, accumulates in the thyroid. Instead of radioiodine, radiometals such as indium-111 (^111^In) may be more relevant for clinical SPECT. However, radiometals cannot be incorporated into the protein directly. Instead, bifunctional chelators are coupled to the protein that promotes radiometal-chelator complex formation. To be successful as an imaging agent, the radiometal-chelator complex must remain intact *in vivo*. Macrocyclic chelators such as DOTA, compared with acyclic chelators such as CHX-A”-DTPA, display higher *in vivo* stability due to a higher binding constant and are favored as a chelator of choice for *in vivo* use (25).

The overall goal of this study was to generate clinically compatible radiolabelling for SPECT imaging of brain Aβ using biAb. The aim was to assess the impact of three different ^111^In-labelling strategies, using acyclic and macrocyclic chelators, on the biAb affinity toward Aβ and mTfR, the *in vivo* brain delivery and interaction with brain Aβ, and the peripheral biodistribution.

## Methods

### Antibody generation

The biAb (RmAb158-scFv8D3) was cloned, expressed and purified by affinity chromatography as previously described (17, 26).

### Chelator conjugation and radiochemistry

Antibody biAb was coupled with 10-fold molar excess of NCS-CHX-A”-DTPA (DTPA), NHS-DOTA (DOTA), or 20-fold molar excess of NHS-TCO combined with DOTA-tetrazine (DOTA-Tz) to facilitate ^111^In-labelling. Conjugation of DTPA to biAb was carried out in 0.1 M boric acid (pH 8.5) at 37°C for 3 h under 350 rpm shaking. DOTA and NHS-TCO were conjugated to biAb in a carbonate buffer, pH 9.2, in room temperature for 1 h under 600 rpm shaking. Uncoupled chelators were removed using NAP5 (GE Healthcare) or Zeba 7K (ThermoFisher) columns, and coupled products were eluted in 0.2 M ammonium acetate buffer pH 5.5.

For the two first radiolabelling strategies, ^111^In-labelling was carried out in 0.2 M ammonium acetate buffer pH 5.5 according to the following parameters: DTPA-biAb and DOTA-biAb, 200–300 μg (0.95–1.43 nmol), were incubated with 50–100 MBq of ^111^In (Curipharm) in a final reaction volume of 500 μL. Labelling reaction with DTPA-biAb was carried out at 37°C for 1 h, and DOTA-biAb was labelled overnight at 37° Reacted products were eluted in PBS using a NAP5 column. These reactions produced [^111^In]DTPA-biAb and [^111^In]DOTA-biAb, respectively.

**Figure 1:**
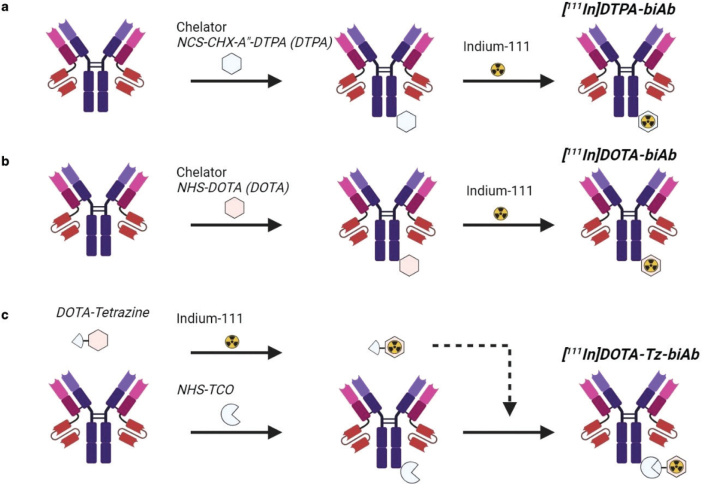
(a) Generation of [^111^In]DTPA-biAb using chelator NCS-CHX-A”-DTPA. (b) Generation of [^111^In]DOTA-biAb using chelator NHS-DOTA. C. Generation of [^111^In]DOTA-Tz-biAb by TCO modification of the biAb followed by the addition of an indium-111 radiolabelled DOTA-tetrazine. Figure was generated in BioRender.

For the third labelling strategy, DOTA-Tz, 12 μg, was incubated with 70 MBq ^111^In for 20 min at 60°C, cooled to room temperature and then incubated for 10 min at room temperature with 300 μg of TCO-biAb, then eluted in PBS using a NAP5 column. This reaction resulted in [^111^In]DOTA-Tz-biAb. A schematic illustration of the three different radiolabelling strategies is shown in Figure 1.

All reaction buffers were stored for >24 h in 1% (w/v) chelex100 (Sigma). Radiochemical yield was determined with instant thin-layer chromatography (iTLC). Radioproducts were applied to silica-gel iTLC strips and 0.2 M citric acid or 0.05 M HCl were used as mobile phases.

### Post-labelling quality control

Labelling stability was determined with EDTA challenge. A total of 500-fold molar excess of EDTA was applied to NAP5 purified ^111^In-labelled biAb, and labelling stability was examined for 72 h. Labelling stability was quantified with iTLC and expressed as percent ^111^In in chelator following EDTA incubation.

ELISA was used to assess mTfR and Aβ binding. A 96-well plate (Corning) was coated with 50 nM synthetic Aβ42 protofibrils (Innovagen, Lund, Sweden) or 1 μg/mL mTfR (in house produced) and incubated at +4°C overnight. Next, blocking was performed with ELISA blocking buffer (PBS, 1% BSA) for 2 h at room temperature. A 50 nM serial dilution of ^111^In-labelled and unmodified biAb was incubated in ELISA buffer (PBS, 0.1% BSA, 0.05% Tween-20) for 2 h at room temperature. Subsequently, peroxidase-conjugated goat-anti mouse IgG, F(ab’)_2_ (Jackson) was added and incubated at room temperature for 1 h. ELISA was developed with K blue aqueous TMB solution and read at 450 nm.

### Animals

Four-month-old wild-type (*n* = 12), 18-month-old wild-type (*n* = 13), and 18-month-old tg-ArcSwe mice (*n =* 12) harboring the human Aβ protein precursor (AβPP) with the Swedish (KM670/671NL) and the Artic (E693) mutations were used in this study. Mice were kept on a C57bl6 genetic background. All procedures in this study were carried out in accordance with the regulations of the Swedish Animal Welfare Agency and complied with the European Communities Council Directive of 22 September 2010 (2010/63/EU) and were approved by the regional Uppsala County Animal Ethics board (5.8.18–35570/2017 and 5.8.18 20401/2020).

### Ex vivo studies and SPECT imaging

To explore the early biodistribution and brain uptake, the three variants of radiolabelled biAb were administered to 4-month-old wild-type mice under light anesthesia using 2% isoflurane. At 2 h after i.v. injection of the radiolabelled biAb, the animal was deeply anesthetised with isoflurane (3–4% in air). A terminal blood sample was obtained from the heart, and plasma was isolated by centrifugation. Animals were then transcardially perfused with 0.9% saline to remove blood. Brains and major organs were isolated, with brains separated into cerebrum and cerebellum and frozen.

Tg-ArcSwe and wild-type mice, aged 18 months, selected for SPECT imaging, were i.v. administered under light isoflurane anesthesia with radiolabelled biAb (Table 1). Blood (8 μL) was sampled from the tail vein at 1, 4, 24, and 48 h post-injection to investigate the blood pharmacokinetics of the different [^111^In]biAbs. At 3 days post-injection, mice were anesthetized with 3% sevoflurane and SPECT-scanned in the prone position using a small animal nanoScan SPECT/CT (Mediso Medical Imaging System, Hungary). The whole mouse was SPECT-scanned with an acquisition time frame of 60 s, resulting in a total scanning time of approximately 60 min. SPECT data were reconstructed as kBq/mL in Nuclide 2.03 software and Tera-Tomo™ 3D SPECT reconstructive algorithm (Mediso Medical Imaging System, Hungary), with 48 iterations into a static image. Following SPECT, CT was performed with a 50-kilovoltage peak X-ray peak, 600 μA, and 480 projections. CT images were reconstructed using filtered back projections. Attained SPECT/CT images were visualized in AMIDE v 1.0.4 (27). Following SPECT and CT, animals were immediately euthanized as described above, and blood, plasma, brain, and major organs were isolated accordingly.

**Table 1:** Administered activity (MBq) and dose (g/kg) of [^111^In]DTPA-biAb, [^111^In]DOTA-biAb and [^111^In]DOTA-Tz-biAb in tg-ArcSwe and wild-type mice.

	Tg-ArcSwe mice		Wild-type mice	
Antibody	Activity (MBq)	Dose (mg/kg)	Activity (MBq)	Dose (mg/kg)
*[^111^In]DTPA-biAb*	5.47 ± 0.35	0.80 ± 0.01	8.35 ± 0.96	0.74 ± 0.01
*[^111^In]DOTA-biAb*	7.35 ± 0.22	3.33 ± 0.04	8.07 ± 0.38	3.02 ± 0.07
*[^111^In]DOTA-Tz-biAb*	7.92 ± 0.13	3.46 ± 0.17	7.57 ± 0.04	2.80 ± 0.04

Values are mean ± SD.

Activity in the blood samples, brain parts, organs, and plasma was measured in a gamma counter (Wizard™, Wallac Oy, Turku, Finland). Retained antibody was quantified as a percentage of injected dose per gram tissue (%ID/g).

### Autoradiography

Following SPECT scanning and isolation of brain tissue, spatial distribution of the three [^111^In]biAb variants was analyzed with autoradiography. Coronal brain cryosections, 20 μm, and a radioactive standard were exposed to phosphor imaging plates (PerkinElmer) for 2 weeks and scanned in typhoon phosphoimager (General Electrics) with a 20 μm resolution. Radioactivity distribution in brain sections was visualized in ImageJ using a royal lookup table. Obtained images were normalized to injected radioactivity dose.

### Statistics

Statistical analysis was performed using the Prism 6 software (GraphPad Software, Inc., La Jolla, CA, USA). One-way ANOVA with Tukey’s multiple comparison test was used to test difference in brain concentrations between the three radiolabelled antibodies at 2 h post injection and in different organs at 72 h post injection, while a t-test was used to compare brain retention of each radiolabelled version of the biAb in tg-ArcSwe and WT mice at 72 h. Values are reported as mean and standard deviation (SD). Elimination phase half-life in whole blood was also estimated in Prism using activity concentrations in blood samples obtained at 4–72 h post-injection using nonlinear regression with a one-phase decay model. Confidence intervals (95%) of half-life estimates were compared to determine potential differences between biAb variants.

## Results

### Post-labelling quality control and labelling stability

**Figure 2:**
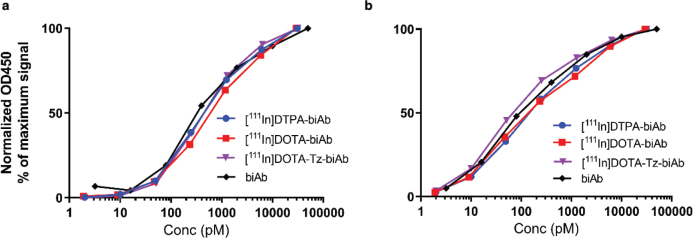
Binding of [^111^In]DTPA-biAb (blue), [^111^In]DOTA-biAb (red), [^111^In]DOTA-Tz-biAb (purple), and biAb (black) to mTfR (a) or Aβ (b) assed by ELISA.

Binding of chelator modified and radiolabelled biAb to mTfR and Aβ was assessed by ELISA. Regardless of radiolabelling strategy, [^111^In]biAb binding to mTfR (Figure 2a) and Aβ (Figure 2b) displayed only minor alterations and reduced affinity compared to non-radiolabelled biAb.

*In vitro* labelling stability of ^111^In was assessed with 500-fold molar excess of EDTA for 72 h. At 24 h, [^111^In]DTPA-biAb and [^111^In]DOTA-Tz-biAb showed near-complete chelation of ^111^In, whereas [^111^In]DOTA-biAb displayed a 15% drop in ^111^In retention. At 72 h, [^111^In]DTPA-biAb and [^111^In]DOTA-biAb showed a 5 and 30% decrease in chelation of ^111^In, respectively, while [^111^In]DOTA-Tz-biAb had complete retention of ^111^In also at this late time point.

### Brain uptake and blood pharmacokinetics

**Figure 3:**
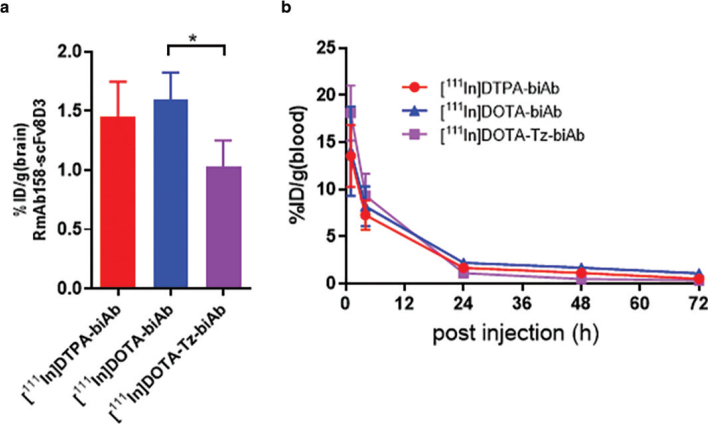
(a) Brain concentrations of [^111^In]DTPA-biAb (*n* = 5), [^111^In]DOTA-biAb (*n* = 4) and [^111^In]DOTA-Tz-biAb (*n* = 3) at 2 h after injection in 4-month-old wild-type mice. (b) Blood concentrations of [^111^In]DTPA-biAb (*n* = 13), [^111^In]DOTA-biAb (*n* = 8) and [^111^In]DOTA-Tz-biAb (*n* = 4) in 18-months-old ArcSwe and aged-matched wild-type mice over 72 h post injection. Values are mean and error bar is standard deviation. ((a) ANOVA, * *P* < 0.05, **P < 0.01, *** *P* < 0.001, *n* = 3–5 per group; B: *n* = 3–5 per group and time point).

Brain uptake at 2 h post-injection was 1.45 ±0.30 %ID/g for [^111^In]DTPA-biAb; 1.60 ±0.23 %ID/g for [^111^In]DOTA-biAb; and 1.03 ±0.23 %ID/g for [^111^In]DOTA-Tz-biAb (Figure 3a). The brain uptake of [^111^In]DOTA-Tz-biAb was somewhat lower than that of the other two biAb variants, but the difference was only significant compared to [^111^In]DOTA-biAb. BiAb half-life in blood was 7.4, 7.0 and 5.6 h for [^111^In]DTPA-biAb, [^111^In]DOTA-biAb and [^111^In]DOTA-Tz-biAb, respectively (Figure 3b). The estimated range of the CI (95%) for the half-life estimates overlapped between the variants.

### SPECT imaging

**Figure 4:**
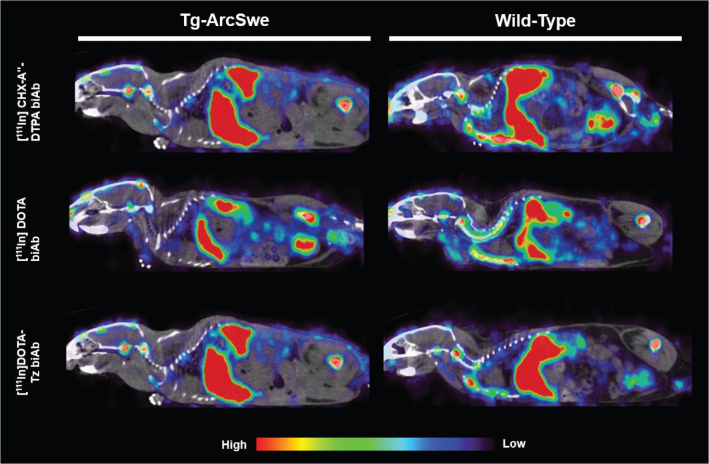
Whole body sagittal SPECT images acquired at 72 h post-injection of [^111^In]DTPA-biAb, [^111^In]DOTA-biAb and [^111^In]DOTA-Tz-biAb in representative tg-ArcSwe and wild-type mice.

SPECT scanning at 72 h after injection of [^111^In]biAb, produced using the three different labelling strategies, revealed a similar organ uptake of all variants, with high radioactive retention in spleen, liver and bones (Figure 4).

### Autoradiography

**Figure 5:**
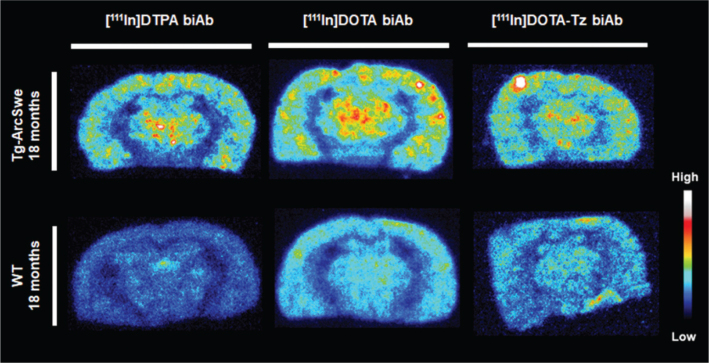
Sagittal representative *ex vivo* brain autoradiography images at 72 h post-injection of [^111^In]DTPA-biAb, [^111^In]DOTA-biAb and [^111^In]DOTA-Tz-biAb in tg-ArcSwe and wild-type mice.

Following SPECT scanning, autoradiography was used to visualize the spatial distribution and brain retention of [^111^In]biAb variants in 20 μm coronal cryosections. The spatial brain distribution was largely similar in tg-ArcSwe and wild-type mice. However, tg-ArcSwe mice displayed somewhat higher radioactivity in areas with Aβ pathology, including cortex, hippocampus and thalamus (Figure 5).

### Brain retention and biodistribution

**Figure 6:**
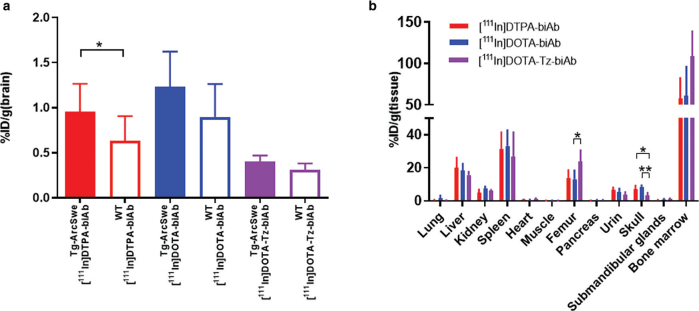
(a) Brain concentrations expressed as %ID/g brain at 72 h in tg-ArcSwe and wild-type mice. (b) Organ biodistribution expressed as %ID/g organ. Values are mean ± SD. WT, wild-type. ((a) T-test between tg-ArcSe and WT for each biAb, * *P* < 0.05, ** *P* < 0.01, *** *P* < 0.001, *n* = 2–7 per group; (b) ANOVA, * *P* < 0.05, ** *P* < 0.01, *** *P* < 0.001, *n* = 4–13 per group).

In line with what was observed with autoradiography, radioactivity measured at 72 h post-injection in brain tissue showed that [^111^In]biAb concentrations tended to be approximately 25–30% higher in tg-ArcSwe compared with wild-type mice but this difference was only significant for the [^111^In]DPTA-biAb variant (Figure 6a). At the same time point, high radioactivity accumulation of all three variants was found in liver, spleen, femur, and bone marrow. [^111^In]DOTA-Tz-biAb displayed higher retention in femur (with bone marrow) and lower retention in skull, compared to the two other variants (Figure 6b).

## Discussion

Antibodies are interesting as PET and SPECT radioligands in AD due to their selectivity and ability to bind to specific forms of Aβ aggregates, some of which are the main targets of therapeutic antibodies presently studied in clinical trials. However, antibodies usually display low and slow delivery into the brain parenchyma. These properties are not suited for diagnostic PET and SPECT radioligands intended for intra-brain targets. The creation of engineered bispecific antibodies targeting endogenous TfR has improved brain uptake via receptor-mediated transcytosis and, thus, presents an attractive option for antibody-based PET- or SPECT-radioligands and immunotherapy

In this study, we radiolabelled biAb, a bispecific antibody targeting mTfR and Aβ, with ^111^In using multiple chelators. Chelator modification and ^111^In-labelling of biAb had no adverse effects on its ability to bind to mTfR and Aβ *in vitro* (Figure 2). *In vivo*, it should be noticed that the blood concentrations of [^111^In]DOTA-Tz-biAb tended to be higher at early time points and lower at later time points compared to the other two variants (Figure 3b). This was also reflected by a slightly faster elimination half-life of [^111^In]DOTA-Tz-biAb, but this difference did not reach significance. Furthermore, the elimination half-life in blood was similar to what has been shown for a radioiodinated version of the biAb ([^125^I]biAb) (23) and other similar bispecific constructs (28).

At 2 h after injection, all chelator variants displayed brain concentrations between 1.0 and 1.5% of injected dose per gram brain tissue (%ID/g) (Figure 3a), similar to previously reported data for [^125^I]biAb (17, 23). However, differences from previous radioiodinated versions appeared at 72 h after injection. When the antibody was labelled with ^111^In, the brain retention in wild-type mice (lacking Aβ pathology) was approximately 10-fold higher than reported for [^125^I]biAb in wild-type mice (17). This indicates a much slower elimination of ^111^In (attached to the antibody or free) from the brain compared to similar studies using ^125^I and ^124^I to radiolabelled biAb.

Despite this high radioactivity retention in wild-type mice, brain retention of ^111^In (attached to the antibody or free) tended to be higher in tg-ArcSwe mice than in wild-type mice (Figure 5 and 6a), demonstrating that radiometal-labelled bispecific antibodies could be used to target brain Aβ. Previous research has also demonstrated high brain uptake of ^111^In-labelled TfR targeting antibodies in wild-type mice (29). In addition, Aβ/TfR bispecific antibodies radiolabelled with zirconium-89 have also demonstrated a relatively high background signal in wild-type mice and a smaller difference in brain retention between Aβ-expressing and wild-type mice compared to radioiodinated versions (30, 31). Multiple mechanisms could explain the prolonged retention of ^111^In (and other radiometals) in the wild-type mouse brain. For example, radiometals are likely to be retained when internalized into a cell, resulting in an extended intrabrain residence time (32). Neurons have been reported to express TfR (33, 34), and the observed prolonged brain retention of [^111^In]biAb could reflect neuronal internalization and retention of ^111^In following the trancytosis of the TfR-binding antibody. Another contributing factor leading to enhanced retention of the biAb in wild-type mice could be glial cell uptake and phagocytosis due to its recognition as a foreign substance. Obviously, these two processes, that is, neuronal and glial uptake, would also be present when radioiodinated biAb is present in the brain. However, in contrast to radiometals, free iodine is a non-residualizing radionuclide and is secreted rather than retained when internalized into a cell.

In the periphery, SPECT imaging and *ex vivo* biodistribution showed similar tissue retention of all ^111^In-labelled variants of biAb, with high retention in the liver, spleen, bone, and bone marrow (Figure 6b). A comparable relative organ retention pattern has been observed with radioiodinated biAb, but at an order of magnitude lower (17). As observed in the brain, the enhanced organ retention could be attributed to the internalization and trapping of ^111^In due to chelator dissociation resulting from intracellular degradation of the antibody. The high liver and spleen retention of ^111^In-labelled biAb may be explained by the activity of phagocytic cells of the mononuclear phagocytic system through binding to the antibodies’ Fc region and subsequent proteolytic degradation (35). SPECT scanning revealed high accumulation in bones containing bone marrow, including the femur, sternum, vertebrae, and skull. The observed bone marrow accumulation of [^111^In]biAb is likely due to the high TfR expression of erythrocyte progenitor cells, leading to TfR interaction, cellular uptake of antibodies, and retention of ^111^In. Dissociation of ^111^In from its chelator could alter biodistribution and reflect *in vivo* distribution of a metal-carrying protein such as transferrin. In our case, this is less likely since blood pharmacokinetics and the half-life in blood of the ^111^In-labelled biAb variants mimic that of radioiodinated biAb, indicating good stability in blood and, thus, accurate quantification of [^111^In]biAb in blood. Additionally, the EDTA challenge demonstrated low transchelation of radiolabelled [^111^In]DTPA-biAb and [^111^In]DOTA-Tz-biAb, thus confirming a stable radiolabelled product. However, DOTA challenged with EDTA resulted in the loss of ^111^In, indicating suboptimal stability. It is unclear why the reduced labelling stability of [^111^In]DOTA-biAb did not translate into an alteration in its *in vivo* distribution. Possible explanations could be a relatively fast mTfR-mediated cell uptake and transport before circulating DOTA loses ^111^In.

In conclusion, we demonstrate that a bispecific antibody engineered to enter the brain at high concentrations could be successfully labelled with ^111^In, retaining TfR-mediated BBB transcytosis and Aβ binding in tg-ArcSwe mice expressing human Aβ. However, prolonged brain retention was observed in age-matched wild-type mice, compared with a radioiodinated version of the biAb, indicating cellular uptake and retention of ^111^In. Further research is needed to pinpoint which types of brain cells internalize biAb and likely contribute to the background signal. The elevated background signal poses a challenge in distinguishing brain tissue containing Aβ from tissue lacking Aβ using *in vivo* SPECT imaging. Prolonged retention of ^111^In was also observed in the periphery, with high uptake and retention in bone containing bone marrow, likely reflecting TfR-mediated uptake, and in organs of the mononuclear phagocytic system (liver and spleen).
